# Low intensity gamma-frequency TMS safely modulates gamma oscillations in probable mild Alzheimer’s dementia: a randomized 2 × 2 crossover pilot study

**DOI:** 10.3389/fneur.2025.1566476

**Published:** 2025-05-15

**Authors:** A. J. Mimenza-Alvarado, S. G. Aguilar-Navarro, I. E. Abarca-Jiménez, I. Vázquez-Villaseñor, Diana I. Luna-Umanzor, C. Dorard, G. Villafuerte

**Affiliations:** ^1^Department of Geriatric Medicine & Neurology, Instituto Nacional de Ciencias Médicas y Nutrición Salvador Zubirán, Mexico City, Mexico; ^2^Escuela Superior de Medicina, Instituto Politécnico Nacional, Mexico City, Mexico; ^3^Actipulse Neuroscience, Inc., Cambridge, MA, United States

**Keywords:** neuromodulation, Alzheimer’s disease, mild cognitive impairment, gamma oscillations, transcranial magnetic stimulation, gamma TMS

## Abstract

**Introduction:**

AD is a progressive neurodegenerative disorder characterized by cognitive decline and memory loss. While traditional treatments targeting beta-amyloid accumulation have shown limited success, there is a pressing need for novel therapeutic approaches. Recent studies have highlighted the role of disrupted gamma oscillations in AD pathology, leading to the exploration of gamma neuromodulation as a potential therapeutic strategy to modify disease progression in individuals with AD dementia. This pilot clinical trial aimed to investigate the electrophysiological effects of low intensity gamma transcranial magnetic stimulation (gTMS) on gamma oscillations in patients with a diagnosis of probable mild AD dementia.

**Methods:**

Employing a randomized, double-blind, sham-controlled, 2 × 2 crossover design, participants underwent a single session of both real low intensity gTMS and sham stimulation. EEG recordings and cognitive assessments were conducted before and after stimulation to assess changes in brain activity and their impact on episodic memory.

**Results:**

We observed statistically significant changes in EEG activity (*n* = 14), indicating transient modulation of gamma oscillations immediately after low intensity gTMS. There was no significant improvement in cognition compared to baseline scores, but we evidenced a positive correlation between electrophysiological changes and cognitive outcome. Importantly, the intervention was well-tolerated, with no significant adverse effects reported.

**Discussion:**

Low intensity gTMS has shown the capability to induce significant changes in brain activity, particularly in gamma oscillations. These findings suggest that low intensity gTMS holds promise as a safe and non-invasive therapeutic approach, challenging the conventional belief that high intensity magnetic pulses are necessary for effective brain modulation. To corroborate these initial findings, further research with extended intervention durations and larger, well-defined cohorts of patients with mild AD dementia is essential. This will validate the potential benefits of low intensity gTMS on cognitive performance in this population.

**Clinical trial registration:**

https://clinicaltrials.gov/study/NCT05784298?term=NCT05784298&rank=1, NCT05784298.

## 1 Introduction

Alzheimer’s disease (AD) is a chronic neurodegenerative disorder characterized by a progressive decline in cognitive function, memory loss, and behavioral changes. As the prevalence of AD continues to rise globally (1), the search for effective therapeutic interventions becomes increasingly vital. Current pharmacological approaches primarily target symptoms and amyloid-*β* accumulation in the brain. While monoclonal antibody therapy shows promise in reducing amyloid-*β* burden in mild cognitive impairment and mild dementia patients (2–4), subgroup analyses reveal variable responses, highlighting the nuanced impact of monoclonal antibody therapy in early AD. Moreover, the potential adverse effects associated with monoclonal antibody therapy require thorough investigation in longer-term studies, emphasizing the ongoing need for non-invasive, non-pharmacological interventions to address cognitive decline and enhance functionality in AD patients.

Mild AD dementia is an early stage of AD characterized by cognitive impairments, including deficits in episodic memory, executive function, language, and visuospatial abilities (5). AD progression is marked by a functional disruption of neural networks in the gamma frequency range, which is believed to play a critical role in higher cognitive processes such as attention, memory formation, and information processing (6, 7). Disruptions in gamma oscillations, typically ranging from 30 to 100 Hz, such as reduced power and synchronization, may contribute to these deficits (8–11). Indeed, restoring gamma oscillations by sensory or optogenetic stimulation in animal studies suggests potential therapeutic benefits (12, 13). Similarly, recent human studies employing non-invasive entrainment of gamma frequency oscillations, such as transcranial alternating current stimulation (tACS), have shown promising physiological changes in network connectivity, hippocampal perfusion, and EEG entrainment, offering further insights into potential treatment modalities (14–17).

Another non-invasive brain stimulation technique, repetitive transcranial magnetic stimulation (rTMS), have been investigated in AD populations, showing positive cognitive outcomes and enhancing memory and neural activity (18–21). TMS utilizes electromagnetic induction to create an electric field within the brain; conventionally, TMS devices employ magnetic fields near 1 tesla to produce electric fields of approximately 100 v/m in the brain. However, current TMS devices are bulky and require expertise for operation, limiting their application to clinical settings. This presents a significant challenge to patients, particularly those with cognitive impairments, since TMS requires long-term interventions (22). A promising solution entails integrating lower intensities into the design of TMS devices, facilitating the development of smaller TMS devices and enabling convenient at-home usage. By employing lower intensities, TMS interventions become safer and more accessible, strengthening adherence and engagement among patients. The conventional approach assumes that high-intensity fields in TMS are necessary for modulating neural activity and consequently eliciting measurable effects on neural oscillations. However, neuromodulation interventions such as tACS, which generate electric fields orders of magnitude smaller to conventional TMS, have been shown to effectively alter the brain’s electrical activity and induce neural oscillations (23–25). In line with this evidence, studies using low intensity TMS have demonstrated its capability to modulate neuronal activity and impact functional connectivity both in pre-clinical models and humans (26–29). Moreover, our recent study demonstrated a safe use of low intensity magnetic fields over 6 months in AD patients (30). These findings suggest that low intensities in TMS protocols also modify brain connectivity and could potentially be used as a therapeutic tool in AD-induced mild dementia.

In this study, we aimed to assess the short-term effects of TMS at low intensity and gamma frequency in a group of patients with a diagnosis of probable mild AD dementia. We hypothesized that low intensity gamma TMS (gTMS) would effectively boost gamma oscillations through modulation of gamma oscillatory power in this cohort. To demonstrate our hypothesis, gTMS was applied in the precuneus, a location involved in a variety of cognitive functions and memory processes important to AD pathogenesis (31). We present the findings of a randomized pilot clinical trial demonstrating an effect on gamma oscillations through the application of a single session of low intensity gTMS. This study explores the relationship between cognitive outcomes and electrophysiological changes following TMS administered at physiological gamma frequencies. We investigate whether low-intensity stimulation can safely modulate electrophysiological activity in individuals with probable mild AD dementia.

## 2 Materials and methods

### 2.1 Study design and sample size calculation

We performed a randomized, double-blind, sham-controlled, and 2×2 crossover pilot study which allows us to control individual differences in response to the intervention and reduce inter-subject variability. The study was conducted at Instituto Nacional de Ciencias Médicas y Nutrición Salvador Zubirán in Mexico, approved by the local ethics committee (CONBIOÉTICA-09-CEI-011-20160627) and registered on the clinicaltrials.gov website (NCT05784298). The study commenced on April 28th, 2022, and was concluded on April 28th, 2023, with an official study completion date, on September 28th, 2023, as recorded on the clinicaltrials.gov website. Written informed consent was obtained for all participants; capacity to consent was ascertained through a legally authorized representative, being a family member in all cases. Patients were accompanied by their representative at all stages of the study.

The sample size for the current study was determined based on the research conducted by Benussi et al. (23), in which a total of 20 participants were included in the final analysis and showed statistical difference in cognitive measurements. We then computed a Cohen’s d value of 0.73 for the calculation, using the Free Statistics Calculators Version 4.0 online tool with an alpha significance level of 0.05 and a statistical power of 0.8 (32). The minimum number of subjects required for a two-way hypothesis was calculated as 18 subjects. To account for the potential non-parametric distribution of the data, this value was then multiplied by 1.2, resulting in 21.6 subjects. Taking into consideration the potential loss of 20% in the sample, the initial sample size of 21.6 subjects was further adjusted. As a result, the study required the recruitment of a total of 27 participants:

### 2.2 Patient selection

Patients aged 65 years or older were eligible to participate if they had an established diagnosis of probable mild dementia due to AD ascertained by a collaborative assessment between a consulting geriatrician/neurologist and a neuropsychologist, in accordance with the diagnostic standards delineated in the Diagnostic and Statistical Manual of Mental Disorders, Fifth Edition (DSM-5) and the criteria stipulated by the National Institute of Neurological and Communicative Disorders and Stroke—Alzheimer’s Disease and Related Disorders Association (NINCDS-ADRDA). The diagnostic process included cognitive assessments guided by the DSM-5 criteria for AD dementia, the criteria of MCI, and tools such as the Montreal Cognitive Assessment (MoCA), and functional evaluations using the Lawton & Brody and Katz indices.

Participants of both sexes were included in the study if they met additional eligibility criteria: a Clinical Dementia Rating (CDR) score of 0.5–1, an Alzheimer’s Disease Assessment Scale-Cognitive Subscale (ADAS-Cog) higher than 15, and full independence in daily living based on the Katz Index of Independence in Activities of Daily Living. Subjects were not included in the study if they showed evidence of depression, as determined by the Geriatric Depression Scale (GDS), if they had uncontrolled medical conditions, anxiety or other psychiatric disorders, metallic implants, a history of seizures, or previous use of any brain stimulation devices. Candidates who were undergoing pharmacological treatment were eligible to participate, provided that their medication regimen had remained consistent for at least 12 weeks prior to the start of the intervention. However, the introduction of new medications and/or cognitive interventions after the first session of intervention was not permitted.

### 2.3 Randomization and masking

Twenty-seven participants were initially screened, with 5 participants not meeting the inclusion criteria and one consented but withdrawn from study prior to randomization. A total of 21 participants were randomly assigned to determine the sequence of interventions. The randomization process was executed using Microsoft Excel: in Column A, a sequential series of patient identifiers (EA001–EA0021) was input, and Column B was employed to assign the interventions using the formula *= RANDBETWEEN(0,1)*, with 0 representing sham stimulation and 1 indicating real stimulation. Randomization was carried out by an independent researcher who had knowledge of the low intensity gTMS device settings for both sham and real stimulation but was not involved in the study itself. Importantly, this information was held in strict confidence and was not disclosed to the researchers responsible for administering the intervention and conducting clinical evaluations. Throughout the study, the unblinded researcher provided the necessary settings for each participant, thereby ensuring that the researchers overseeing the study remained unaware of the specific intervention administered to each patient. The possibility of unblinding from the participants’ perspective was low since low intensity magnetic stimulation does not elicit somatosensory activation nor an auditory cue; after both sessions concluded, patients were asked in which session they received the real stimulation and only 10 out of 21 patients guessed correctly. This approach helped maintain the study’s integrity and minimized potential biases.

### 2.4 Intervention

The device utilized in this study was developed and manufactured by Actipulse Neuroscience (Boston, MA, United States). The custom-made circular coil with a 50 mm diameter featured a central hole to accommodate an EEG electrode (Supplementary Figure S1) and was positioned beneath the flexible EEG cap and surrounding the Pz electrode to target the precuneus. The device operates by delivering electric current through the coil to generate a rapidly changing magnetic field at a gamma frequency of 40 Hz and an approximate magnitude of 100 gauss. COMSOL Multiphysics^®^ modeling software (Burlington, MA, United States) was employed to calculate this intensity to achieve an induced electric field of 1 V/m at 3 cm from the coil (Supplementary Video S1).

### 2.5 Trial procedures

**Figure 1:**
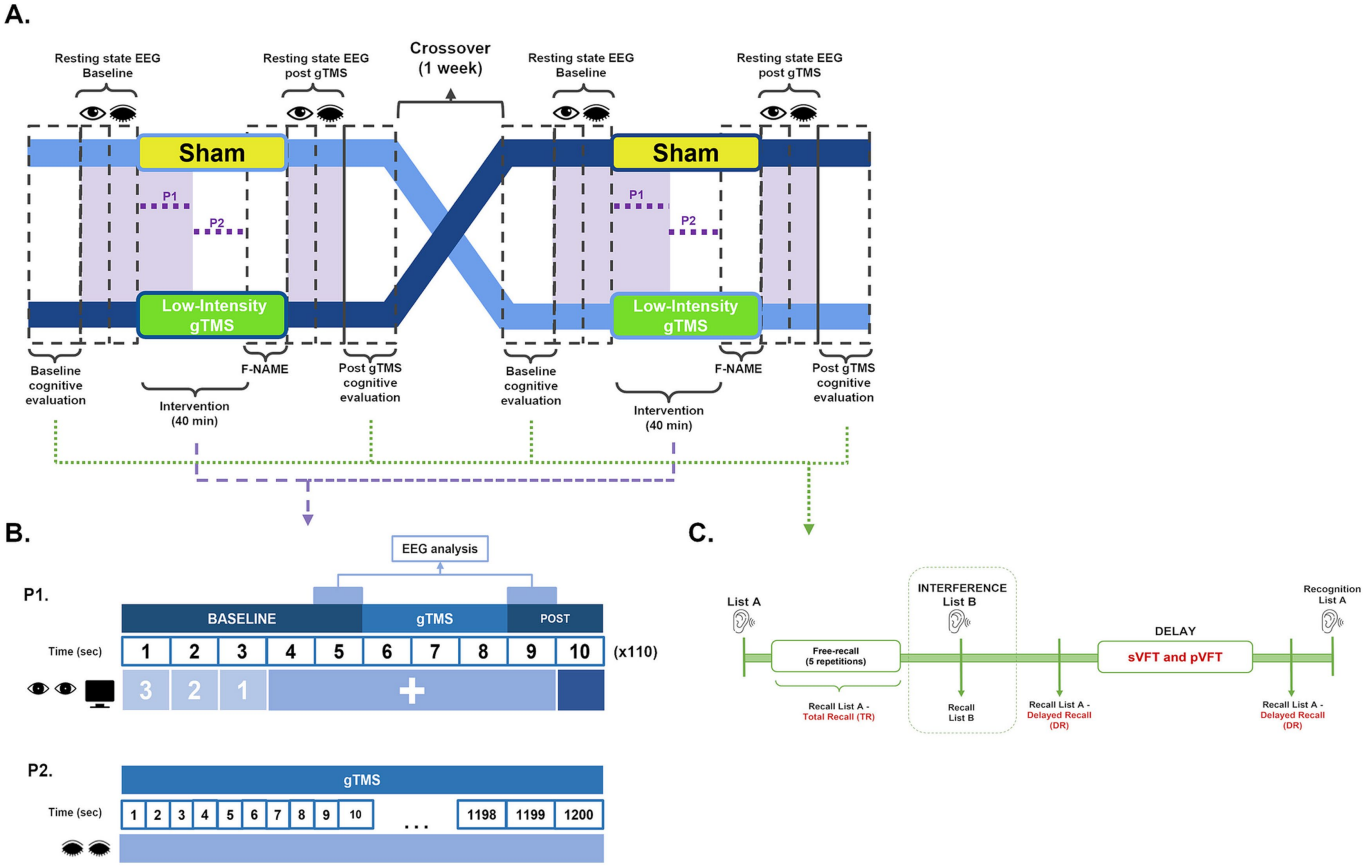
Study design. **(A)** The study employed a randomized, double-blind, sham-controlled, 2 × 2 crossover design, comprising two sessions with a one-week wash out interval between them. In the first session, participants were randomly assigned to either the sham or low intensity gTMS stimulation group. The session started with a comprehensive baseline cognitive evaluation, consisting of RAVLT, sVFT, and pVFT as depicted in the diagram. Following the cognitive assessment, EEG recordings were conducted during a 2.5 min baseline resting state with open eyes and another 2.5 min with closed eyes. Subsequently, either sham or real low intensity gTMS sessions started for 40 min. F-NAME test was performed at the end of the full session, before resting state EEG recordings post-intervention. Finally, another cognitive evaluation was performed, following the same protocol as the baseline assessment. A crossover was conducted for the second session, where participants switched stimulation arms. Both the baseline and post-cognitive assessments, as well as the intervention and resting state EEG recordings, followed the same design as described for the first session. Times at which EEG recordings were performed are highlighted in purple. **(B)** Paradigm P1 consisted of 110 bursts of 10 s each, given at 40 Hz for 20 min. During P1, participants were engaged with a screen throughout the session. Each burst commenced with a countdown from 3 to 1, preparing participants for the upcoming activity. Following the countdown, a “+” sign remained on the screen for the subsequent 6 s, providing a consistent visual cue. As the final second approached, the screen transitioned into complete darkness. EEG was recorded throughout P1. Paradigm P2 involved prolonged gTMS at 40 Hz for 20 min, during which no EEG was recorded. **(C)** For the RAVLT, participants heard a list of 15 nouns (List A) and recalled as many words as possible after each of five free-recall trials. An interference trial followed, in which participants heard a new list (List B) and recalled as many words as possible. Total Recall (TR) was assessed by summing the number of words recalled across the five free-recall trials of List A. Delayed recall (DR) of List A was tested after the interference and delay periods, followed by a final recognition assessment. To evaluate verbal fluency, both sVFT and pVFT were administered during the RAVLT delay period.

Each participant underwent both a real low intensity gTMS session lasting 40 min and a sham stimulation session (without emission of magnetic pulses from the coil) of the same duration (Figure 1A). A washout period of 1 week was used between crossover of sessions to minimize carryover effects and reduce inter-individual variability (23, 33–35). Following each session, participants were interviewed for any potential adverse effects (AE) and information was documented and collected at the Memory Clinic of the Instituto Nacional de Ciencias Médicas y Nutrición Salvador Zubirán.

Each session employed two distinct paradigms designed to assess different aspects of low intensity gTMS effects. The first paradigm P1 consisted of applying a total of 110 bursts of 3 s at 40 Hz during 20 min. P1 was immediately followed by the second paradigm P2, involving a continuous 40 Hz stimulation lasting for another 20 min (Figure 1B).

### 2.6 Outcome measures

#### 2.6.1 EEG

For each session, EEG recordings were conducted at three distinct time points: resting state prior to stimulation (2.5 min, eyes open, with participants instructed to fixate on a static cross displayed on the screen; followed by 2.5 min, eyes closed), during P1 (eyes open, Figure 1A), and resting state after P2 (2.5 min, eyes open, with participants instructed to fixate on a static cross displayed on the screen; followed by 2.5 min, eyes closed) (Figure 1A). To accurately timestamp the initiation of stimulation during the EEG recording of P1, an external trigger (EMOTIV Extender) was synchronized with the stimulator, generating marking events at the start of stimulation. During all sessions, patients were exposed to white noise through headphones to avoid environmental noise interfering with the EEG signal acquisition. EEG data recording was performed with the EMOTIV PRO software and the EMOTIV EPOC Flex EEG system (EMOTIV^®^, San Francisco, United States), equipped with 32 saline electrodes affixed to participants’ heads using a flexible cap (EasyCap^®^, Herrsching, Germany), and their locations were configured based on the international 10–20 system (36) (Supplementary Figure S1). To ensure optimal data quality, the impedance of the electrodes was consistently maintained below 20 kΩ throughout all experimental phases. The EEG data was sampled at a rate of 1,024 Hz, initially filtered within the range of 0.2 Hz to 45 Hz, and subsequently downsampled to 128 Hz.

#### 2.6.2 Cognitive assessment

Each session consisted of a baseline and post-stimulation cognitive assessment including Rey Auditory Verbal Learning test (RAVLT), semantic Verbal Fluency Test (sVFT) and phonemic Verbal Fluency Test (pVFT) (Figure 1C) (37, 38). While RAVLT evaluates verbal episodic memory and is designed as a list-learning paradigm, VFT are short tests that assess the ability to retrieve specific information within restricted search parameters to evaluate the effect of the intervention on information retrieval from memory through verbal fluency.

Application of the RAVLT consisted in the subject hearing a list of 15 different nouns (List A) and being asked to recall as many words as possible after each of 5 repetitions of free recall. This was followed by an interference trial, where the subject heard a second list of nouns (interference List B) and was asked to recall as many as possible. Total recall (TR) was then assessed by asking the subject to recall as many words as possible from List A. A TR score was obtained by adding all recalled words from the 5 free-recall repetitions. To avoid learning effects in RAVLT, different lists of words were used for the baseline and post stimulation evaluation.

sVFT and pVFT were performed during the delay period of RAVLT, during which participants had to generate a list of words that shared a semantic feature for the sVFT (animals or fruits) and a list of words that began with a single letter (“F” or “L”), each list different from pre- and post-stimulation.

At the end of P2, a cross-modal associative memory test, known as the Face-Name Associative Memory Test (F-NAME) (39, 40), was performed (Figure 1A). The task consisted of three phases: practice, encoding, and retrieval. During practice, participants received instructions and an example. In the encoding phase, they viewed 12 unfamiliar faces paired with names and judged if each name suited the face. In the retrieval phase, participants completed three tests: identifying a familiar face among three options, recalling the first letter of a name when shown a face, and selecting the correct name from three options for a given face. The researcher provided verbal instructions and guided participants through each phase to ensure clarity.

### 2.7 EEG data processing and analysis

#### 2.7.1 Phantom control and exclusion strategy based on phantom control EEG data

A ballistic head was created based on a previously described protocol (41) to use as phantom control and identify noises inherent to the low intensity gTMS device. Briefly, a 3D model of the head was printed using a Creality K1 Max 3D Printer and Hyper Pla 3D Printing Filament (Creality, Shenzhen, China) and a gelatin ballistic head was made using this mold. Before performing the sham and real low intensity gTMS recordings, the ballistic head was thoroughly cleaned with an alcohol moistened cotton swab to remove impurities and prevent interference with the EEG signal. Subsequently, the stimulation coil was placed beneath the flexible EEG cap, as described for the patients. The EEG ground electrodes were placed on the ears of the ballistics head using wooden sticks to prevent them from falling off or moving. Once the set up was properly positioned, both sham and real low intensity gTMS sessions were applied to the ballistic head in a randomized manner, and eighteen sham and eighteen real low intensity gTMS EEG data were recorded. Visual assessment of EEG recordings allowed data exclusion where the stimulation artifact was not detected because of underlying noise and the same approach was applied to the patients’ EEG recordings (Supplementary Figure S2). The source of this underlying noise remains unclear, but we hypothesize that it may have been caused by an unknown interaction between the magnetic fields and the Bluetooth data transmission of the EEG system. Although the entire setup was powered by batteries instead of an AC power source, the noise persisted unpredictably and exclusively during real stimulation conditions in both phantom and human participant recordings. Given that this noise-related artifact affected the entire recording and masked the stimulation signal, we decided to exclude the full recording rather than rejecting individual trials.

#### 2.7.2 EEG data pre-processing for patient and phantom recordings

The open-source MNE-Python package for analysis of human neurophysiological data (42, 43), and the NumPy (44) and Matplotlib (45) libraries were used for the data preprocessing of the phantom and patients’ EEG recordings. The following preprocessing steps were applied to all recordings: First, an Independent Component Analysis (ICA) model consisting of 31 components was fitted to the raw EEG data. The resulting components were visually inspected as time-series and spatial maps to identify and exclude components representing eye-blinking and cardiac activity artifacts. Furthermore, all 32 channels were carefully examined for excessive noise or artifacts, and if any were present, the channels were interpolated using MNE’s internal functions. The data was then subjected to a high-pass filter (1 Hz) and a low-pass filter (45 Hz), followed by re-referencing to the common average.

For the P1 stimulation EEG recordings, a specific time window was excised from the data to eliminate artifacts caused by the TMS pulses. This window comprised 200 ms before stimulation, the entire 3,000 ms of stimulation, and 200 ms after stimulation. Two distinct epochs were then created and concatenated for subsequent analysis: a baseline epoch, spanning from 1,000 ms before stimulation to 200 ms before stimulation, and a period of interest epoch, encompassing data from 200 ms after stimulation to 1,000 ms after stimulation (Figure 1B). Another round of ICA was performed on the concatenated epochs, this time with 23 components, to identify and remove components containing artifacts resulting from the concatenation of epochs. Finally, all epochs were manually inspected to identify and discard any remaining epochs of poor quality. The epoched data was processed for time-frequency analysis.

For the baseline and post-stimulation EEG recordings during eyes open and eyes closed conditions, arbitrary events were created every five seconds of recording, and epochs from −1.5 to 1.5 s relative to each event were used for spectral analysis.

#### 2.7.3 Pre-processed EEG data analysis for patient and phantom recordings

For EEG data analysis from P 1, the preprocessed epochs from real and sham conditions were used to calculate the time frequency representation of each epoch using Morlet wavelets. The number of cycles per wavelength was always one half of the analyzed frequency (1 to 40 Hz). For each subject, the average time frequency representation was calculated and then these individual averages were used to create a grand average for each condition. To evaluate differences between conditions, regions of interest in the time frequency plane were selected beforehand. Four different regions of interest were defined for each electrode depending on the time of interest (between 0.5 s after stimulation and 0.9 s after stimulation) and the activity in theta (4–8 Hz), alpha (9–12 Hz), beta (13–30 Hz) and gamma (31–40 Hz.). All values were normalized using a logratio method to baseline (the period between 0.9 s and 0.5 s before stimulation). For each of the 32 electrodes, the average activity of each of the 4 ROI was calculated and then compared between conditions. A summary of the excluded components and interpolated channels is provided in Supplementary Table S1.

#### 2.7.4 Baseline and post resting state EEG

For each preprocessed epoch of both conditions (sham and real) at both time points (baseline and post stimulation) power spectral density was computed using python’s MNE implementation of multitaper method. Individual PSD representations of epochs were then averaged within subjects and then a grand average for each condition was created. Then, we calculated the power activity of the grand average in the canonical frequencies mentioned above; to compare between conditions the power activity post intervention was normalized using a logratio method to the baseline power activity (baseline recording) (46).

### 2.8 Statistical analysis

#### 2.8.1 EEG patient and phantom data

A Wilcoxon signed-rank test was performed between conditions for all electrodes. For each of the 32 electrodes, values were normalized using a logratio method to baseline and the average activity of each of the 4 ROI was calculated and then compared between conditions. Due to the high number of comparisons, *p* value correction for multiple comparison using False Detection Rate (FDR) was used (47). The effect size *r* was calculated for the EEG power measures displaying significant differences when compared to the sham condition; 95% confidence intervals (CI) were calculated to establish its precision.

#### 2.8.2 Clinical and cognitive data

The statistical analysis was conducted using Python and a one-way ANCOVA to compare the post-intervention scores between the real and sham groups, adjusting for baseline scores, the order of the intervention and the groups. This analysis controlled initial differences, providing a more accurate assessment of the intervention effects. As there was no baseline measurement for F-NAME test scores (face, letter, name and total) paired *T*-tests were used to assess differences between sham and low intensity gTMS.

#### 2.8.3 Correlation EEG and cognitive tests

Correlations between cognitive test outcomes and the activity of electrodes that showed a statistically significant change in power due to gTMS were examined using Spearman’s rank correlation coefficient. Scatter plots were generated to visualize these correlations, and descriptive statistics including means, standard deviations, medians, interquartile ranges (IQR), and Shapiro–Wilk tests for normality were computed for all variables of interest.

## 3 Results

**Figure 2:**
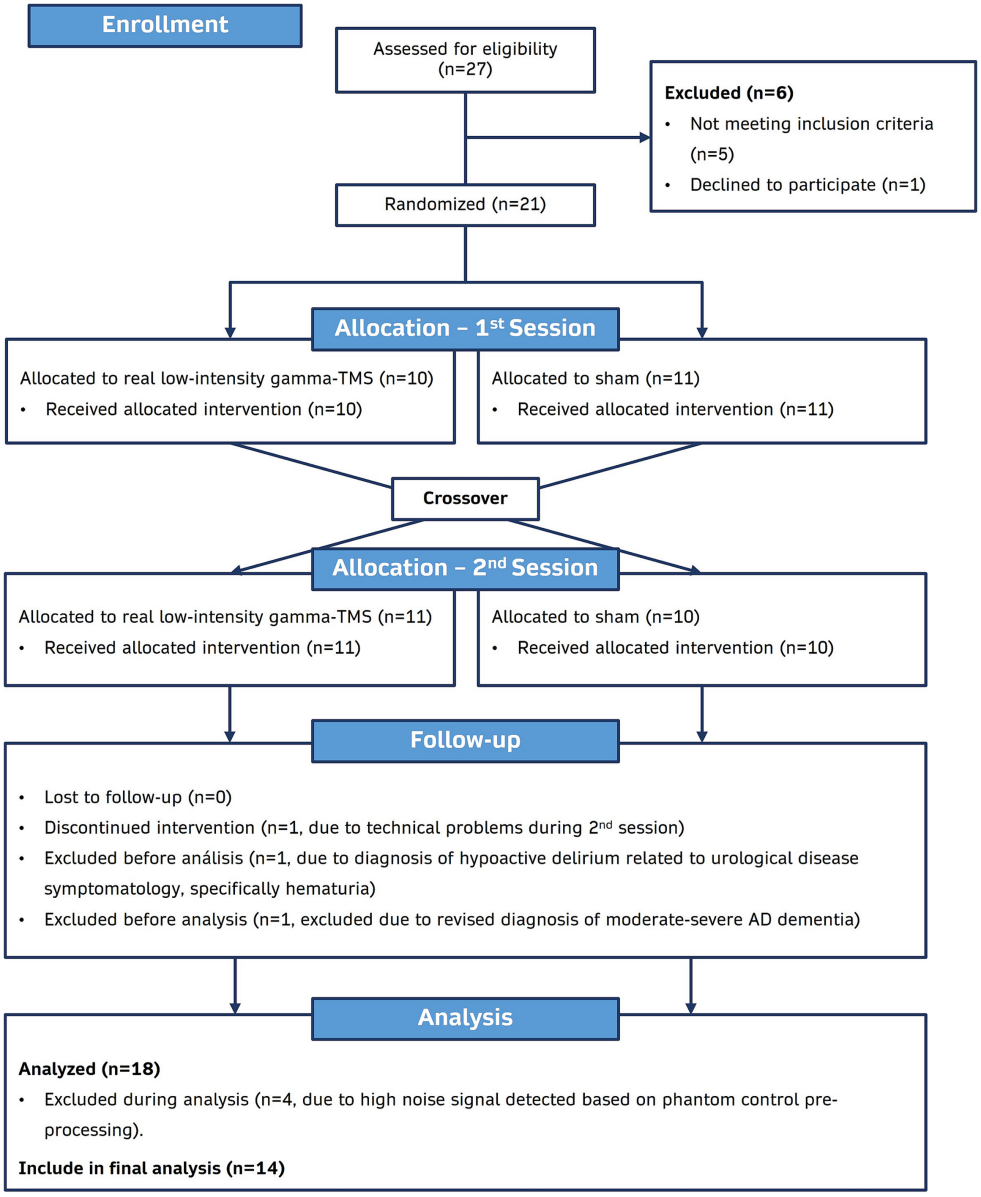
CONSORT flow diagram of study participants. Randomization, trial-group assignment, and follow-up in the trial.

Of the 21 participants with probable mild AD dementia who were enrolled and randomized for this study, 18 completed the study, and 14 were included in the final analysis (Figure 2). The four participants excluded from the analysis presented a high noise signal detected with the phantom pre-processing, which could have biased the analysis (Supplementary Figure S2). Demographic and clinical information for included participants is presented in Table 1.

**Table 1:** Demographic and clinical characteristics of patients.

Variable		Values (*n* = 14)
Age, years		77.1 ± 6.1
Sex, female		43%
Education, years		14.6 ± 4.9
MoCA total score		14.8 ± 4.7
ADAS-Cog total score		27.4 ± 8.1
Katz ADL Index		5.9 ± 0.5
Global CDR score	**0.5**	2 (14.3%)
	**1.0**	12 (85.7%)
GDS score		2.5 ± 2.5

MoCA, Montreal Cognitive Assessment; ADAS-Cog, Alzheimer’s Disease Assessment Scale-Cognitive Subscale; KATZ ADL, Katz Index of Independence in Activities of Daily Living; CDR, Clinical Dementia Rating Scale; GDS, Geriatric Depression Scale. All values, except for sex and global CDR score, are expressed as mean ± SD. Global CDR score is expressed as *n* (percentage). *N* = 14. Global CDR Score values: 0.5 - very mild dementia; 1.0 - mild dementia.

Out of the 18 participants that completed the intervention, 13 (72%) experienced no adverse effects. One participant reported a headache 1 day after the first session (sham), with no apparent triggers or factors that could have worsened the condition. To alleviate the symptoms, the participant was prescribed a non-steroidal anti-inflammatory drug and showed a significant improvement in symptoms during the follow-up period. Another participant experienced a headache after the first session (real low intensity gTMS), as well as headache and nausea following the second session (sham). While the symptoms of the first case were alleviated after taking oral pain relief medication, the symptoms persisted for a period of 2 weeks after the second session. Overall, the intervention was well-tolerated by most participants, with only two cases (11%) of minor and unrelated adverse effects reported, demonstrating the safety of gTMS with intensities markedly lower than the typical 10,000 gauss utilized in classic TMS protocols.

### 3.1 Low intensity gTMS bursts induce transient changes in gamma band oscillations during stimulation in patients with probable mild AD dementia

**Figure 3:**
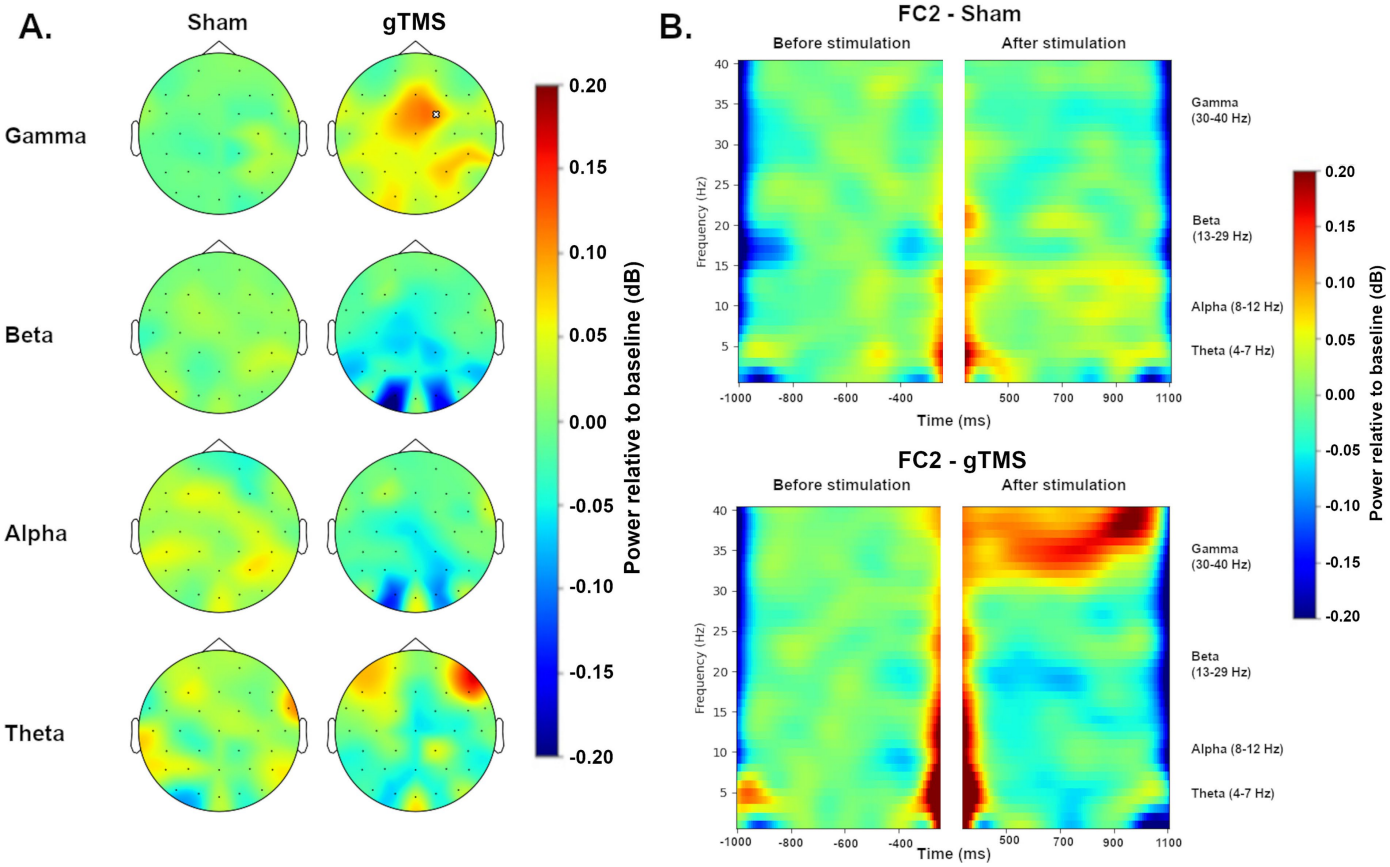
Spectral analysis conducted following low intensity gTMS revealed significant alterations in electrophysiological activity. **(A)** Topographic maps showing localization and direction of significant changes in the average power of all bands, which occurred immediately after low intensity gTMS, as compared to sham stimulation. The electrode FC2, exhibiting a significant increase in gamma power relative to baseline (dB), is indicated with an “x.” **(B)** Time-frequency maps of FC2 electrode exhibiting a significant difference in average power within the gamma frequency band compared to sham. *N* = 14.

We firstly focused on acute alterations in brain activity immediately after a burst of low intensity gTMS in comparison to sham stimulation. In the P1 paradigm, EEG were recorded before and after each burst of gTMS (Figure 1B) to perform time-frequency analyses and allow the examination of the immediate after-effects and short-term electrophysiological changes induced by low intensity gTMS. Interestingly, significant changes in gamma power were observed in EEG recordings immediately after low intensity gTMS compared to sham stimulation (Figure 3A). These changes were located at the frontal region of the brain, while the gTMS was applied on the precuneus. Specifically, the low intensity gTMS group exhibited a significant increase in gamma power at the frontal electrode FC2 (*p =* 0.0002, *q =* 0.031) (Figure 3B). The differences in alpha, beta and theta frequencies did not reach statistical significance for any of the 32 electrodes. The effect size *r* for the changes in FC2 was calculated to be −0.86, indicating a large effect according to Cohen’s (48) conventions. The 95% confidence interval for the effect size ranged from −1.38 to −0.34, reinforcing the statistical effect observed for the FC2 electrode upon gTMS. Hence, our results suggest that gTMS affect gamma oscillations in patients with a diagnosis of probable mild AD dementia.

### 3.2 Low intensity gTMS over a long duration does not induce brain electrophysiological changes

We also aimed to investigate the sustained effects of low intensity gTMS over a longer duration, providing insights into potential long-lasting changes. To do so, we assessed electrophysiological changes in brain activity by EEG resting state recordings before (baseline) and after (post) low intensity gTMS or sham stimulation, with both eyes open and closed, to avoid modulation of alpha and theta waves. We compared EEG recordings at the end of the full session, including the two paradigms P1 and P2, corresponding to 20 min gTMS bursts and 20 min continuous gTMS, respectively, (40 min in total), with the baseline for each region of interest: theta (4–8 Hz), alpha (9–12 Hz), beta (13–30 Hz) and gamma (31–40 Hz). When assessing differences with sham stimulation, we did not observe significant changes in power across all frequency ranges after low intensity gTMS (Supplementary Figure S3).

### 3.3 One session of low intensity gTMS does not improve cognitive function in patients with probable mild dementia due to AD

To assess whether a single session of low intensity gTMS had a similar effect on cognition, we set as a secondary endpoint the evaluation of changes in episodic memory following P1–P2 session of low intensity gTMS versus sham stimulation, measured by the RAVLT, the sVFT and pVFT, and the F-NAME association task. To account for variability in baseline scores and the order of the intervention, a one-way ANCOVA was performed, controlling for these covariates to better assess the potential effects of the intervention. The ANCOVA results demonstrated that baseline scores were significant predictors of post-intervention performance on the RAVLT (*R*^2^ score = 0.536, *β* = 0.845, *p* < 0.0001), sVFT (*R*^2^ score = 0.306, *β* = 0.6069, *p* = 0.004), and pVFT (*R*^2^ score = 0.575, *β* = 0.7306, *p* < 0.0001) (Supplementary Table S2). However, the effects of group (real low-intensity gTMS vs. sham) and intervention order were not statistically significant for any of the measures (RAVLT – group: *β* = 0.068, *p* = 0.974 and order: *β* = −0.2208, *p* = 0.916; sVFT – group: *β* = 0.140, *p* = 0.939 and order: *β* = −1.4616, *p* = 0.430; pVFT – group: *β* = 0.3819, *p* = 0.722 and order: *β* = 0.7498, *p* = 0.489). Controlling baseline scores, the analysis showed that pre-existing cognitive abilities influenced outcomes more strongly than the intervention itself. Additionally, the analysis of the F-NAME association task using a paired *t*-test yielded similar results, confirming no significant changes in final scores observed during low intensity gTMS compared to sham (Supplementary Table S2).

### 3.4 gTMS-induced changes in gamma power at FC2 electrode correlate with an improvement in cognitive outcomes

**Figure 4:**
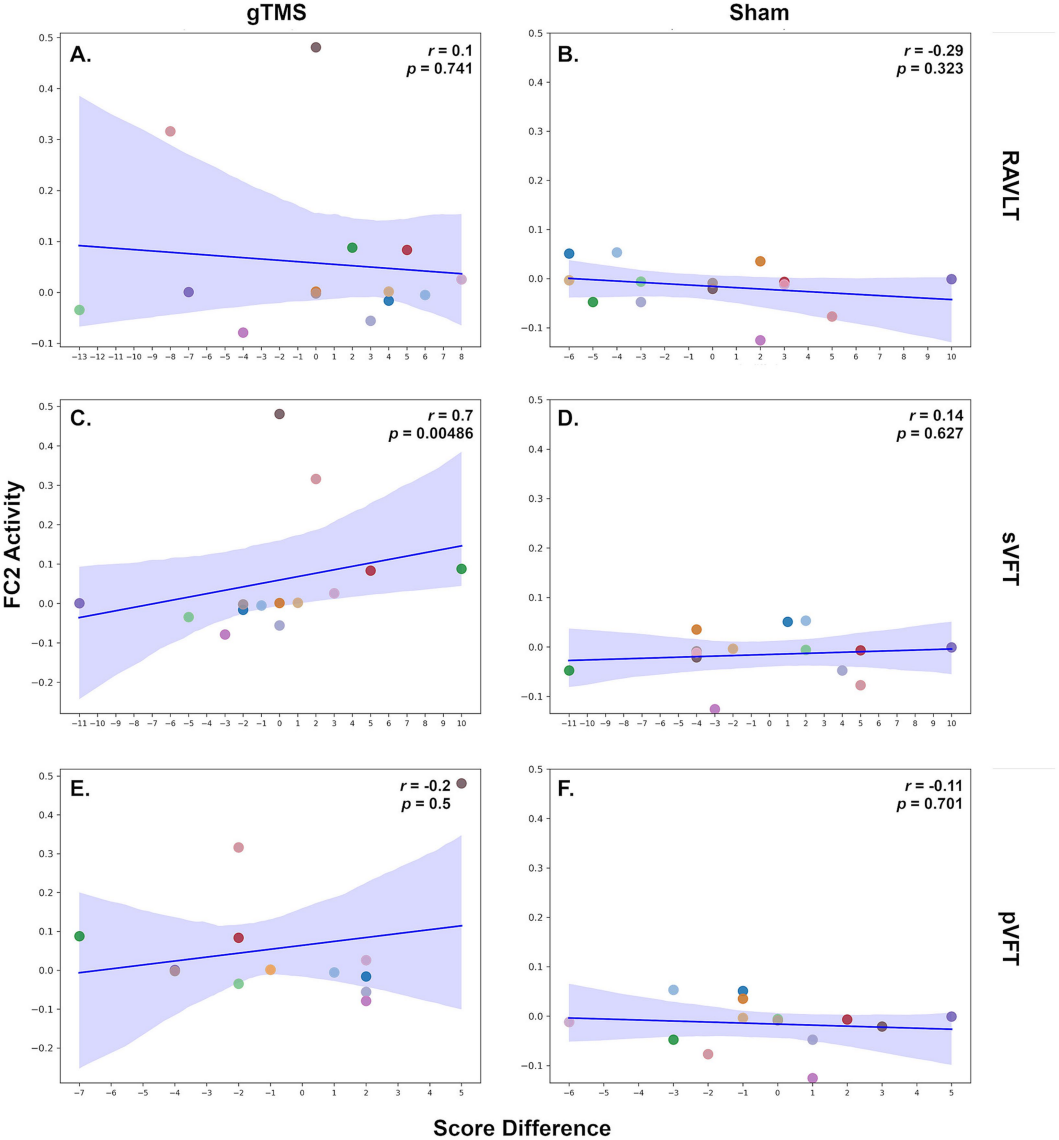
Relationships between cognitive performance metrics and FC2 changes post low intensity gTMS in patients with mild dementia due to AD. Scatter plots illustrating the relationships between FC2 activity and the cognitive tests RAVLT, sVFT, and pVFT. Panels **(A,C,E)** depict the correlations after a real low intensity gTMS session, while panels **(B,D,F)** show the correlations after a sham session. A positive correlation is observed between sVFT and FC2 electrode (Spearman’s *r* = 0.7, *p* = 0.00486).

Since we observed significant changes in gamma oscillations in FC2 after short-term gTMS, we aimed to explore the relationship between these results and cognitive functions after stimulation. Spearman’s correlation analyses revealed a significant positive correlation between changes in sVFT scores and FC2 gamma activity after low intensity gTMS (Spearman’s *r* = 0.7, *p* = 0.00486) (Figure 4). However, no significant correlations were observed between changes in RAVLT or pVFT scores and FC2 gamma activity in either the sham or real low intensity gTMS conditions (RAVLT real: *r* = 0.1, *p =* 0.741; RAVLT sham: *r* = −0.29, *p* = 0.323; sVFT sham: *r* = 0.14, *p* = 0.627; pVFT real: *r* = −0.2, *p* = 0.5; pVFT sham: *r* = −0.11, *p* = 0.701).

## 4 Discussion

In this study, we explored the effects of low intensity gTMS on EEG activity and cognitive function in patients with a diagnosis of probable mild AD dementia. Our results revealed transitory electrophysiological changes in cortical areas, specifically in the gamma frequency range at FC2 electrode. Although an increase in gamma activity positively correlated with higher sVFT scores, no statistically significant cognitive differences were observed between the groups. The intervention also proved to be safe, with only two participants reporting headaches, which were transient and resolved with standard treatment. These findings suggest that low intensity gTMS directly influences brain activity, inducing significant changes in gamma oscillations that may correlate with cognitive function in patients with probable mild AD dementia.

Gamma neuromodulation, a non-invasive technique stimulating brain activity at 40 Hz, shows promise in treating mild AD dementia, with several studies having explored its effects on enhancing cognitive function and underlying AD pathology (14, 15, 18, 23–25). The findings reported in this manuscript provide evidence of immediate gamma modulation following a single stimulation session of low intensity gTMS in patients with probable mild AD dementia. This is, to our knowledge, the first report in which low intensity TMS modulates gamma oscillatory power in a clinical population. These results are consistent with previous studies that have demonstrated the efficacy of other neuromodulation techniques, such as tACS (49, 50), on inducing neural oscillations and altering electrical brain activity using weak oscillating electric currents.

The precuneus, a key brain region involved in multiple cognitive functions such as memory, self-awareness, visuospatial processing, executive functions, and consciousness (51), has been identified as a crucial hub in AD-related neural networks (18, 19, 21, 31). Prior research, such as Koch et al. (19), has shown that high-frequency rTMS targeting the precuneus enhances cortical excitability, strengthens functional connectivity with medial frontal areas, and improves episodic memory in early-stage AD patients.

Our findings align with these studies, demonstrating modulation of gamma oscillations at FC2 following low-intensity gTMS applied to the precuneus. The differences in oscillatory activity changes between our study and Koch et al. (19) may reflect distinct neurophysiological mechanisms driven by varying stimulation intensities and frequencies. While Koch et al. reported increased beta oscillations, associated with large-scale network communication and memory processes within the default mode network (52, 53), our study observed gamma oscillations, which are linked to local cortical processing and cognitive functions like attention and working memory (54–56).

These results suggest that low-intensity gTMS targeting the precuneus can modulate frontal cortical dynamics, with gamma oscillations reflecting frequency-dependent neuromodulation. Previous studies using tACS and rTMS have shown that gamma-range stimulation enhances local cortical excitability and network dynamics, particularly in conditions like AD, where gamma activity is often disrupted (18, 23, 24). The distinction between beta and gamma modulation highlights the complexity of network-specific responses to TMS, suggesting that different stimulation protocols may preferentially engage distinct oscillatory mechanisms.

Although initial statistical analysis identified multiple significant electrodes, only FC2 remained statistically significant after applying FDR correction to control for Type I errors. This suggests that while the effects of low-intensity gTMS may be more widespread, our limited sample size could have restricted the detection of broader network-level effects.

The modulatory effect seen on gamma oscillations at FC2 was found to be transient. The observed modifications did not maintain their presence during post-stimulation resting state assessments, indicating that, while gTMS at low intensities possesses the capacity to influence brain activity, achieving enduring electrophysiological effects may require repeated low intensity gTMS sessions and extended treatment periods for long-lasting changes. Consistently, low intensity gTMS might also entail several sessions for notable cognitive improvements to manifest. Previous research exploring the manipulation of gamma oscillations in AD patients using rTMS has shown promising outcomes with prolonged intervention durations. For instance, sustained gamma modulation and enhanced cognitive function were observed after an 8-week, 12-session gamma rTMS protocol targeting the left temporoparietal cortex (25). Similarly, another study reported significant positive effects on episodic memory following a 10-day gTMS intervention targeting the precuneus (18). Another phase 2 clinical trial, consisting in 24-week treatment with a 2-week intensive course with rTMS followed by a 22-week maintenance phase in patients with mild-to-moderate AD, demonstrated a slowdown of cognitive and functional decline (57). While extended treatment regimens may pose challenges for individuals with mild AD dementia, one advantage of low intensity gTMS lies in its potential to facilitate the development of compact, user-friendly devices that could eventually be managed by patients or their caregivers at home. Furthermore, the utilization of low intensities contributes to the safety of the intervention by decreasing potential adverse effects.

Despite the observed electrophysiological changes, there was no impact of low intensity gTMS on cognitive performance. For RAVLT, sVFT, and pVFT, baseline scores were robust predictors of post-intervention performance, whereas group assignment (low intensity gTMS vs. sham) and intervention order did not significantly impact the results. This finding underlines the importance of considering individual variability in cognitive abilities when evaluating the effectiveness of interventions like low intensity gTMS. Our approach employed two separate paradigms to capture different effects of low-intensity gTMS, including both immediate electrophysiological responses and longer-lasting cognitive effects after the 40-min stimulation. We acknowledge that the consecutive application of these two paradigms could have led to saturation or counteraction effects, potentially masking the individual effects of each paradigm. Future studies should also consider modifying this approach to better understand the individual and combined effects of these paradigms on brain activity and cognition.

Interestingly, although no significant improvement in sVFT scores was observed after the intervention, a positive correlation between changes in sVFT scores and FC2 gamma activity was found. This suggests that gamma oscillations in FC2 may reflect neural processes involved in semantic memory retrieval. However, it is important to note that this correlation does not imply direct therapeutic potential. The absence of similar correlations with the RAVLT and pVFT suggests that low-intensity gTMS may have task- or domain-specific effects on brain networks. Therefore, further research is needed to determine whether these effects directly influence cognitive performance or merely reflect cognitive state.

We acknowledge several limitations in our study that may affect the generalizability of our results. First, the absence of amyloid-*β* and tau biomarkers to complement clinical evaluations in diagnosing mild AD dementia represents a significant limitation. Despite the use of standardized clinical assessments for diagnostic purposes within our cohort, the lack of biomarkers complicates the establishment of a definitive mild AD diagnosis, contributing to a sample with high heterogeneity. Variability in participants’ initial cognitive abilities may have influenced their responsiveness to the low intensity gTMS intervention. Although we included baseline cognitive scores as covariates in our statistical analysis, the relatively small cohort size may have underpowered the study, potentially impacting the robustness of our results.

Second, we recognize the potential influence of microsaccades on gamma band EEG effects. While we employed ICA to remove eye-blink components, small eye movements, such as microsaccades, might still contribute to the observed gamma band activity. Microsaccades are small, involuntary eye movements that occur during fixation and can generate saccadic spike potentials (SPs), which have temporal and spectral characteristics similar to induced gamma band responses (iGBRs) (58, 59). SPs can produce broadband gamma activity that may be misinterpreted as neural gamma oscillations. While we carefully reviewed our data for residual artefacts related to eye movements, the possibility of subtle contributions from microsaccades cannot be entirely ruled out.

Third, we acknowledge limitations due to our EEG system’s filtering parameters. The system samples at 1,024 Hz but is automatically downsampled to 128 Hz, applying a 0.2–45 Hz filter, which attenuates activity above 45 Hz. Although we conducted an additional analysis (Supplementary Figure S4) with a 0.2–64 Hz filter, frequencies beyond 45 Hz remain suppressed due to built-in filtering. While our data reliably capture activity within 0.2–45 Hz, this limitation raises the possibility that the observed gamma effects could reflect a broader shift in the broadband power spectrum, as discussed by Donoghue et al. (60). Exploration of higher frequency bands (e.g., >80 Hz) would be needed to rule out this confound and better characterize spectral changes induced by low-intensity gTMS.

Additionally, the exclusion of data due to a noise-related artifact, likely arising from an unknown interaction between the magnetic fields and the Bluetooth data transmission of the EEG system, along with the notable difference between conditions in the number of excluded components during preprocessing (Supplementary Table S1), may have influenced the output data. To our knowledge, there is currently no optimized or validated preprocessing strategy to effectively address artifacts arising from the interaction between TMS and wireless data transmission. Consequently, our methodology and findings should be interpreted with this limitation in mind.

In conclusion, this study provides compelling neurophysiological evidence of gamma oscillation modulation through a single session of low intensity gTMS targeting the precuneus region in patients with a diagnosis of probable mild AD dementia. These findings challenge the prevailing paradigm that high-intensity magnetic pulses are necessary to modulate brain activity, highlighting the potential of low-intensity approaches for therapeutic modulation. The favorable safety profile of low-intensity gTMS further supports its suitability for long-duration interventions, offering a safer, more accessible option for dementia treatment.

While our study focused on identifying acute electroencephalographic alterations following a single stimulation session, the transient nature of these effects supports the need for future research to explore multi-session protocols. Investigating the sustainability and long-term impacts of low-intensity gTMS on both brain activity and cognitive function will be critical to establishing its therapeutic potential. Such studies could pave the way for the development of compact, user-friendly devices that patients or caregivers could manage at home, further enhancing the accessibility and practicality of this intervention.

## Data Availability

The raw data supporting the conclusions of this article will be made available by the authors, without undue reservation.

## Supplementary material

SUPPLEMENTARY VIDEO S1 Electric field model. Simulation of the electric field generated 3 cm from the coil. **(A)** The blue sphere represents the participant's head. **(B)** Circular coil positioned surrounding the Pz electrode. **(C)** Exact location of the Pz electrode.

SUPPLEMENTARY FIGURE S1 Low Intensity gTMS and EEG setup. **(A)** A 32-channel EEG cap equipped with 32 saline electrodes affixed to participants' heads using a flexible cap (EasyCap^®^, Herrsching, Germany). The locations of the electrodes were configured based on the international 10–20 system. **(B)** The circular coil with a 50 mm diameter featured a central hole to accommodate an EEG electrode and was positioned beneath the flexible EEG cap and surrounding the Pz electrode to target the medial parietal cortex and the precuneus.

SUPPLEMENTARY FIGURE S2 Phantom control exclusion strategy. **(A)** Phantom EEG recording shows the gTMS signal clearly detectable in the purple box. **(B)** The stimulation signal in the phantom EEG recording is masked by a device-linked artifact. **(C)** If the artifact was present in the patient's EEG recording, the recordings were included in the preprocessing and analysis. **(D)** Example of patient’s EEG recording excluded from the preprocessing and analysis because of the absence of the artifact.

SUPPLEMENTARY FIGURE S3 Resting state spectral analysis. The topographic maps illustrate the spatial distribution and direction of changes in the average power of theta, alpha, beta, and gamma bands during resting state EEG analysis, for both eyes open and eyes closed conditions. Results are represented in colors according to the power relative to baseline (dB).

SUPPLEMENTARY FIGURE S4 Power spectral density (PSD) analysis under different filtering conditions. Panels **(A,C)** show PSD analysis for gTMS and sham conditions, respectively, using the reported pipeline, which includes low-pass filtering. Panels **(B,D)** show PSD analysis for gTMS and sham conditions, respectively, without low-pass filtering. The removal of low-pass filtering does not allow visualization of changes in frequencies above 45 Hz due to the device-intrinsic limitations in bandwidth. Specifically, the EEG system samples at 1,024 Hz but is automatically downsampled to 128 Hz, setting the Nyquist frequency at 64 Hz and applying a 0.2–45 Hz filter. The system also exhibits high attenuation at 50 Hz and 60 Hz, which helps reduce electrical noise but may impact nearby frequencies. As a result, activity above 45 Hz is attenuated, preventing detection of broadband power shifts or high-frequency artifacts.
