# Supplementary figures and images for: Low intensity gamma-frequency TMS safely modulates gamma oscillations in probable mild Alzheimer’s dementia: a randomized 2 × 2 crossover pilot study
Source: Front Neurol. 2025 May 15;16:1566476. doi: 10.3389/fneur.2025.1566476 (PMC12121370; doi:10.3389/fneur.2025.1566476)

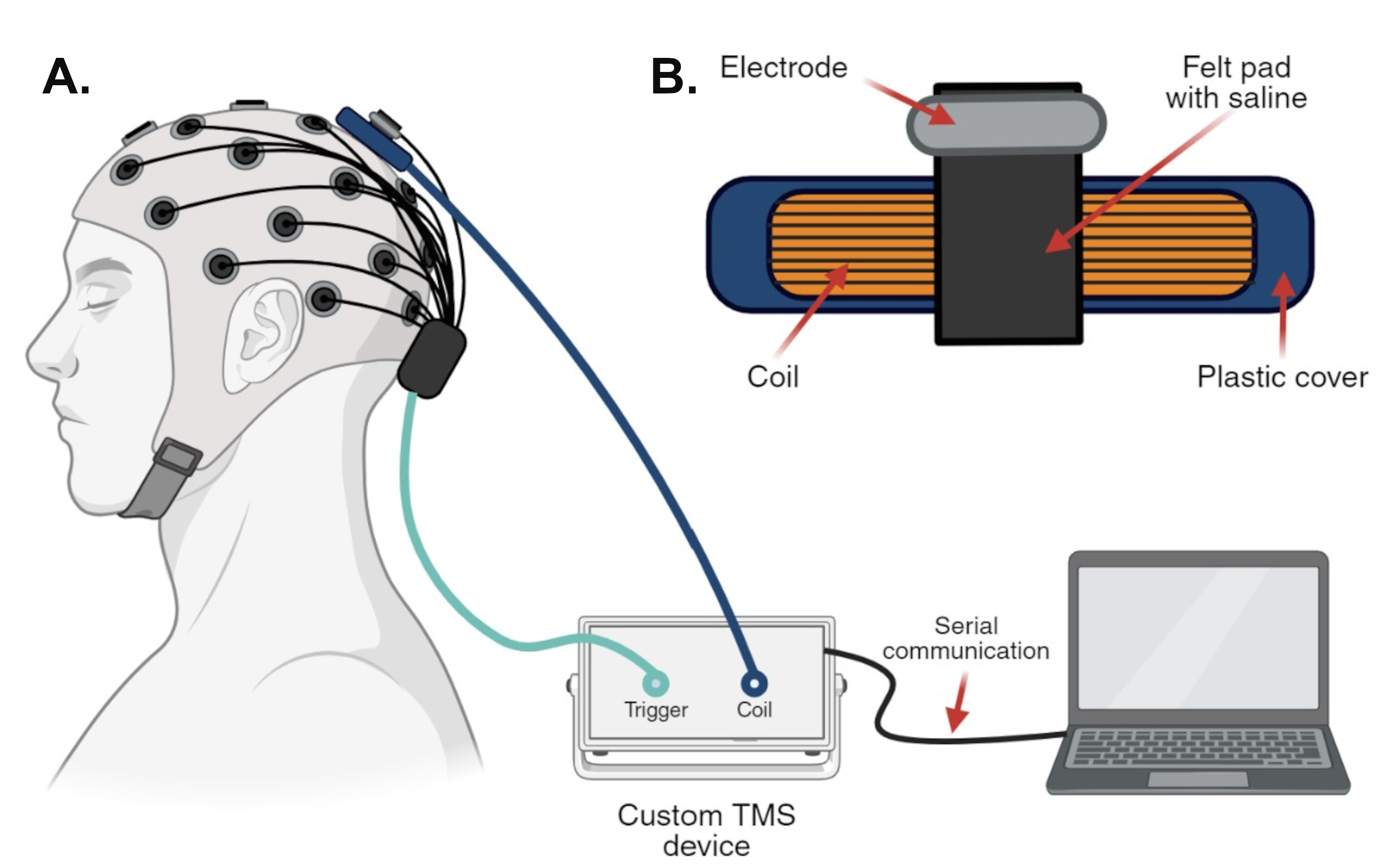

Supplement: SUPPLEMENTARY FIGURE S1 — Low Intensity gTMS and EEG setup. (A) A 32-channel EEG cap equipped with 32 saline electrodes affixed to participants' heads using a flexible cap (EasyCap®, Herrsching, Germany). The locations of the electrodes were configured based on the international 10–20 system. (B) The circular coil with a 50 mm diameter featured a central hole to accommodate an EEG electrode and was positioned beneath the flexible EEG cap and surrounding the Pz electrode to target the medial parietal cortex and the precuneus. [file Image_1.jpeg]

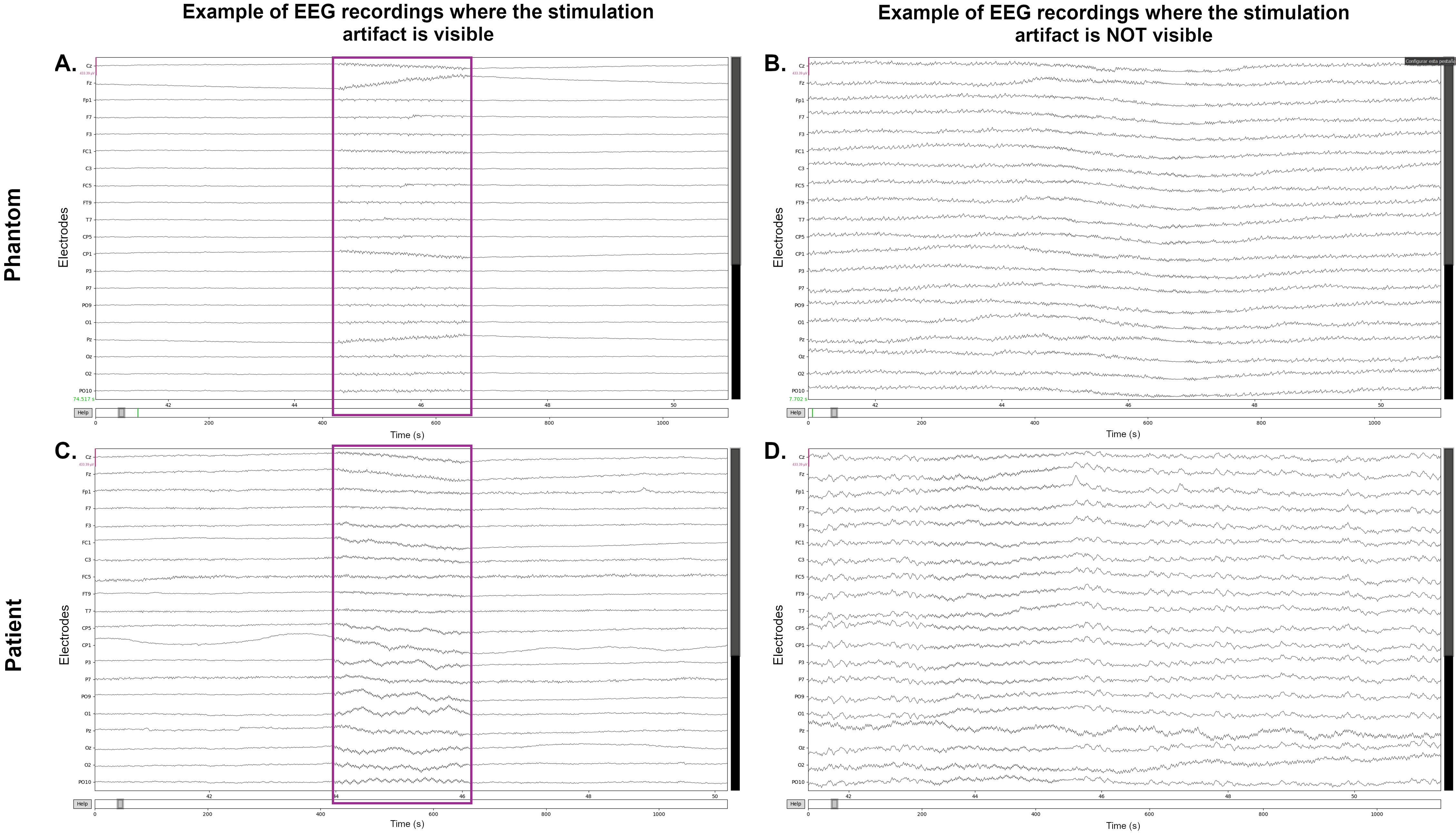

Supplement: SUPPLEMENTARY FIGURE S2 — Phantom control exclusion strategy. (A) Phantom EEG recording shows the gTMS signal clearly detectable in the purple box. (B) The stimulation signal in the phantom EEG recording is masked by a device-linked artifact. (C) If the artifact was present in the patient's EEG recording, the recordings were included in the preprocessing and analysis. (D) Example of patient’s EEG recording excluded from the preprocessing and analysis because of the absence of the artifact. [file Image_2.jpeg]

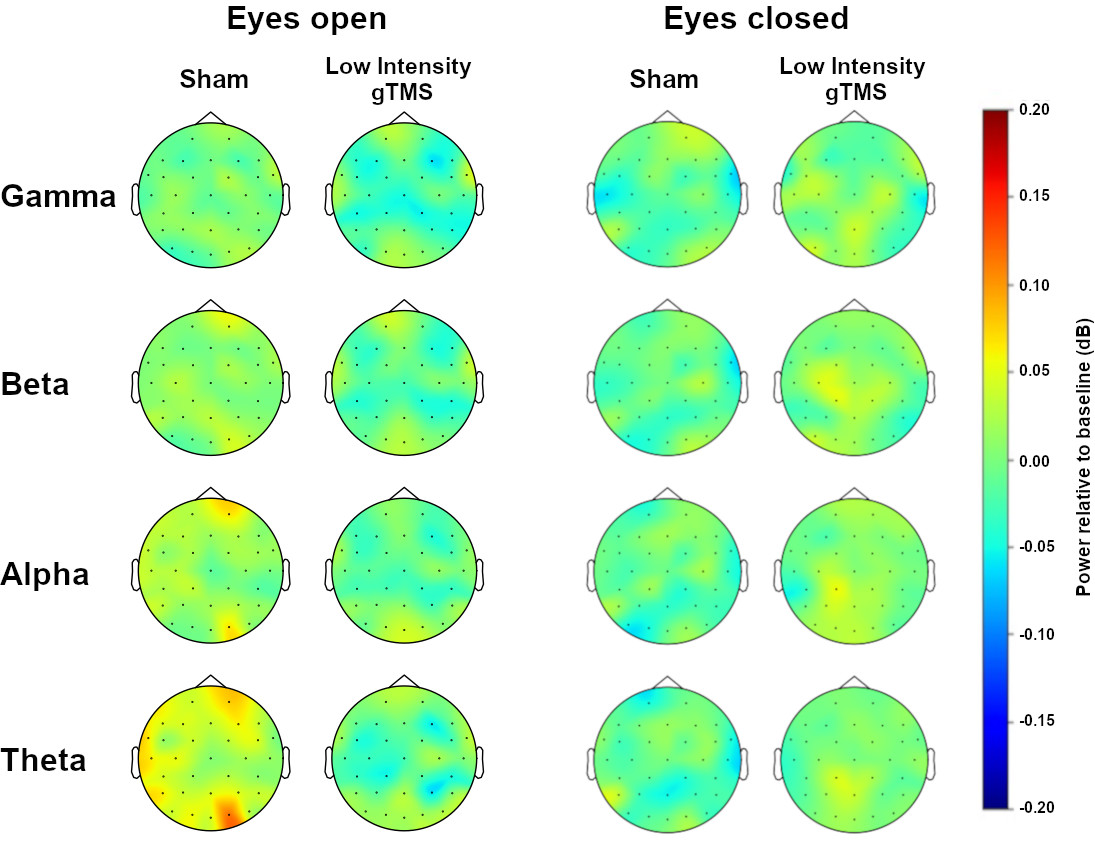

Supplement: SUPPLEMENTARY FIGURE S3 — Resting state spectral analysis. The topographic maps illustrate the spatial distribution and direction of changes in the average power of theta, alpha, beta, and gamma bands during resting state EEG analysis, for both eyes open and eyes closed conditions. Results are represented in colors according to the power relative to baseline (dB). [file Image_3.jpeg]

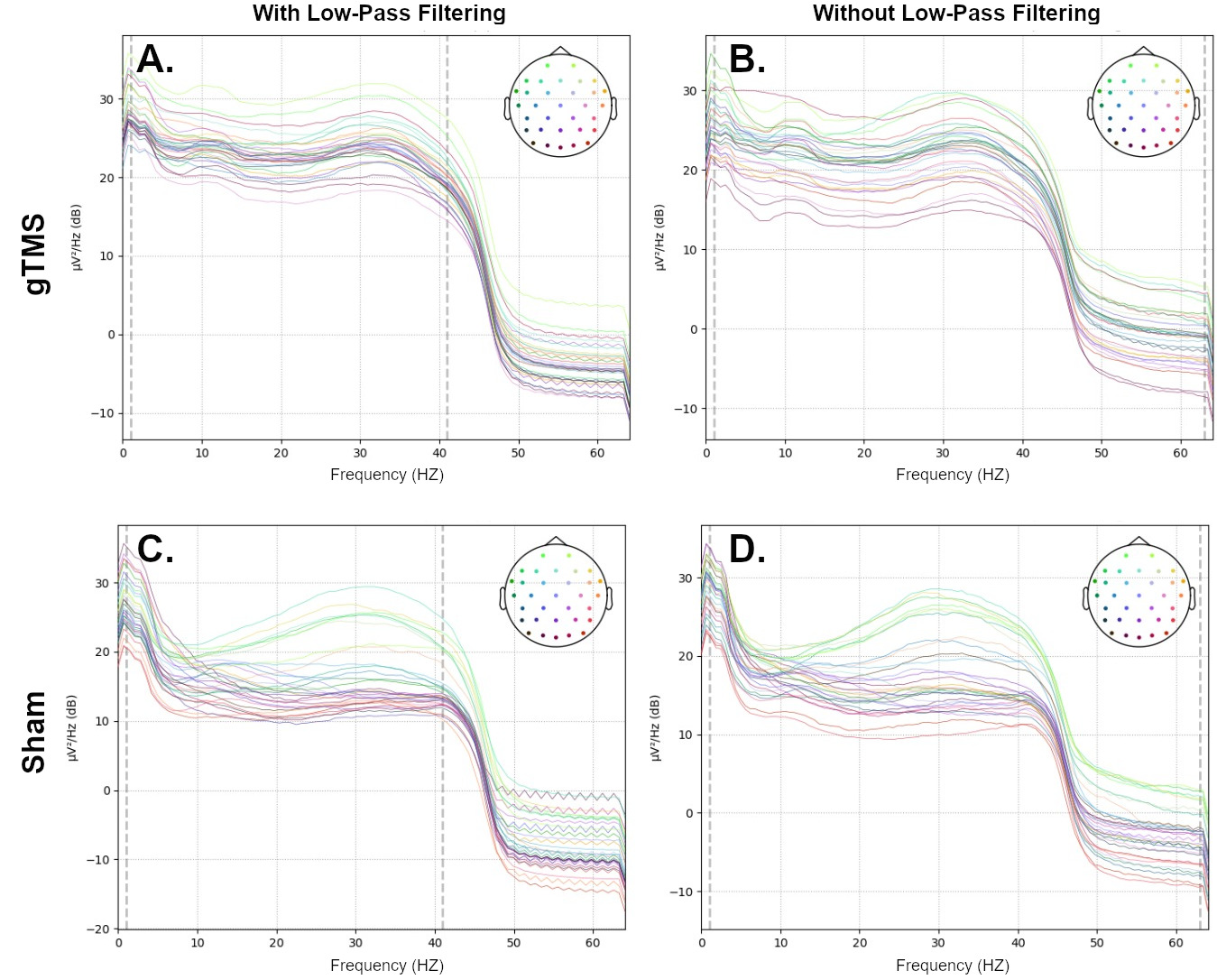

Supplement: SUPPLEMENTARY FIGURE S4 — Power spectral density (PSD) analysis under different filtering conditions. Panels (A,C) show PSD analysis for gTMS and sham conditions, respectively, using the reported pipeline, which includes low-pass filtering. Panels (B,D) show PSD analysis for gTMS and sham conditions, respectively, without low-pass filtering. The removal of low-pass filtering does not allow visualization of changes in frequencies above 45 Hz due to the device-intrinsic limitations in bandwidth. Specifically, the EEG system samples at 1,024 Hz but is automatically downsampled to 128 Hz, setting the Nyquist frequency at 64 Hz and applying a 0.2–45 Hz filter. The system also exhibits high attenuation at 50 Hz and 60 Hz, which helps reduce electrical noise but may impact nearby frequencies. As a result, activity above 45 Hz is attenuated, preventing detection of broadband power shifts or high-frequency artifacts. [file Image_4.jpeg]
