# Supplementary material for: Low intensity gamma-frequency TMS safely modulates gamma oscillations in probable mild Alzheimer’s dementia: a randomized 2 × 2 crossover pilot study
Source: Front Neurol. 2025 May 15;16:1566476. doi: 10.3389/fneur.2025.1566476 (PMC12121370; doi:10.3389/fneur.2025.1566476)
Supplement: Supplementary file 6 [file Data_Sheet_1.pdf]

	PCA 1 (31 components) – Excluded Components			Interpolated Electrodes (32 channels)			PCA 2 (23 components) – Excluded Components			Eliminated Epochs		
	**Mean**	**SD**	**Variance**	**Mean**	**SD**	**Variance**	**Mean**	**SD**	**Variance**	**Mean**	**SD**	**Variance**
**gTMS**	5	2.57	6.62	2	0.92	0.84	8	1.65	2.73	11	5.04	25.41
**Sham**	4.43	2.47	6.11	2.79	1.42	2.03	2.29	1.14	1.30	8.29	4.16	17.30

Table S1. Excluded Components and Interpolated Channels During Pre-Processing. This table summarizes the mean number of excluded components, interpolated electrodes, and eliminated epochs as part of the pre-processing strategy. Abbreviations: gTMS = Low intensity gamma repetitive transcranial magnetic stimulation; SD = standard deviation; IQR = interquartile range; N = 14.
