# Supplementary material for: Low intensity gamma-frequency TMS safely modulates gamma oscillations in probable mild Alzheimer’s dementia: a randomized 2 × 2 crossover pilot study
Source: Front Neurol. 2025 May 15;16:1566476. doi: 10.3389/fneur.2025.1566476 (PMC12121370; doi:10.3389/fneur.2025.1566476)
Supplement: Supplementary file 7 [file Data_Sheet_2.pdf]

	Timepoint	Intervention	Mean (SD)	Median (IQR)	Paired-T Test Statistic (*p* value)
**F-NAME**	**Face**	**gTMS**	9.71 (2.37)	10.5 (3.5)	0.92 (0.374)
		**Sham**	9.29 (2.43)	9.5 (3)	
	**Letter**	**gTMS**	1.86 (1.75)	2 (3)	0.86 (0.405)
		**Sham**	1.36 (1.34)	1 (0)	
	**Name**	**gTMS**	6.29 (1.73)	6.5 (3.25)	0.14 (0.888)
		**Sham**	6.21 (0.97)	6 (0.75)	
	**Total**	**gTMS**	17.86 (3.94)	19 (2.75)	1.26 (0.229)
		**Sham**	16.86 (3.13)	17.5 (3.5)	

Table S2. Cognitive assessment scores. The table presents paired-T tests results for F-NAME scores applied during P2 paradigm. Data shown includes the test statistic and the *p*-value for the F-NAME Face, Letter, Name, and Total scores. gTMS= low intensity gamma repetitive transcranial magnetic stimulation**;** F NAME=face–name associations task; SD=standard deviation; IQR=interquartile range. N=14
